# Effects of Diastolic Blood Pressure on Brain Structures and Cognitive Functions in Middle and Old Ages: Longitudinal Analyses

**DOI:** 10.3390/nu14122464

**Published:** 2022-06-14

**Authors:** Hikaru Takeuchi, Ryuta Kawashima

**Affiliations:** 1 Division of Developmental Cognitive Neuroscience, Institute of Development, Aging and Cancer, IDAC, Tohoku University, 4-1 Seiryo-cho, Aoba-ku, Sendai 980-8575, Japan; ryuta@tohoku.ac.jp; 2 Smart Aging Research Center, Tohoku University, Sendai 980-8575, Japan; 3 Department of Advanced Brain Science, Institute of Development, Aging and Cancer, Tohoku University, Sendai 980-8575, Japan

**Keywords:** hypertension, brain structures, dementia, cognitive functions, longitudinal

## Abstract

Hypertension is a pervasive public health concern due to strong associations with cardiovascular diseases and stroke. Alternatively, the associations between hypertension and the risk of Alzheimer’s disease are complex and recent large sample studies reported positive associations. In this paper, we examine the associations between diastolic blood pressure (BP) and subsequent changes in brain structure and cognitive function over several years by multiple regression analyses (with adjustment for a wide range of potential confounding variables) among a large cohort from the UK Biobank. Higher baseline diastolic BP was associated with a slightly smaller relative increase (relative improvements) in reaction time and a slightly greater reduction in depression scores. Higher baseline diastolic BP was also associated with a greater total gray matter volume (GMV) retention, while aging alone was associated with GMV reduction. White matter microstructural analyses revealed that a greater diastolic BP was associated with reduced longitudinal mean and regional fractional anisotropy, greater increases in mean and regional mean diffusivity, radial diffusivity, and axial diffusivity, a greater decline in mean intracellular volume fraction, and greater increases in mean and regional isotropic volume fraction. These white matter microstructural changes were consistent with those seen in the aging process. Additional analyses revealed a greater cheese intake level at baseline, which is associated with a subsequent decline in diastolic BP and a relative subsequent increase in depressive tendency together with a relative increase in fluid intelligence and visuospatial memory performance. These results are congruent with the view that a higher BP in the aging brain has a complex role.

## 1. Introduction

Hypertension is a primary risk factor for cerebral infarction [1]. Hypertension has also been shown to cause ischemic or hemorrhagic brain damage in the white and gray matter [2]. However, the relationship between hypertension and neurological diseases is complex, and in old age, low blood pressure (hypotension) rather than hypertension is thought to be a risk factor for Alzheimer’s disease [3]. It has also been suggested that low BP is a pre-clinical symptom rather than a cause of dementia [4]. However, a recent Mendelian randomization study reported that high blood pressure is causally linked to a lower risk of dementia [5].

Numerous studies have examined the relationships between hypertension and various cognitive functions in the elderly. Three large-sample (N = 1134−5680) longitudinal studies of aging adults found that hypertension leads to greater declines in executive function, visuospatial motor skill, delayed recall test performance, word fluency, and memory performance [6]. Similarly, several large-sample cross-sectional studies have reported relationships between hypertension and greater age-related declines in cognitive function [6]. A review of BP that summarizes a wide range of cross-sectional studies of the associations between BP and cognitive functions revealed complex associations between BP and cognitive functions. It also showed that higher BP is associated with negative cognitive outcomes during middle age and that moderately high BP is associated with better cognitive outcomes at older age [7].

A wide range of brain imaging studies have been conducted to investigate the relationship between hypertension and altered neural mechanisms. Hypertension has been associated with a subsequent decrease in resting blood flow [8] and with an increase in current and subsequent WM lesions [9]. A diffusion tensor imaging (DTI) study by Rosano et al. [10] found that higher blood pressure in early old age was associated with reduced fractional anisotropy (FA) and greater mean diffusivity (MD) 20 years later, while lower FA and greater MD are characteristics of aging and dementia and thought to be markers of cerebral degeneration/damage/structural loss [11,12]. However, that study did not examine the relationship between FA and MD changes. A similar relationship between FA and high blood pressure was found in a cross-sectional study [13]. In addition, a recent cross-sectional study using UK biobank data [14] revealed the associations of higher BP with lower FA, higher MD, as well as lower intracellular volume fraction (ICVF, which reflects neurite compartment density) and higher isotropic volume fraction (ISOVF, which reflects extracellular free water diffusion), which are measured by neurite orientation dispersion and density imaging (NODDI). These cross-sectional findings support the view that a higher BP is associated with compromised white matter integrity. Further, a meta-analysis of cross-sectional studies concluded that hypertension is associated with reduced hippocampal volume [15]. However, most longitudinal studies on the relationships between hypertension and regional brain volumes are of limited statistical power due to sample sizes [15].

Some diets can lower BP. Among these, *lactobacilli*, including those of certain types of cheese, are associated with decreased BP [16]. Additionally, a recent middle-sized sample study showed that a greater cheese intake is associated with lower BP [17], probably because peptides with angiotensin-converting enzyme (ACE, which narrows blood vessels and increases BP)-inhibiting or BP-lowering activity have been identified in many cheeses [18]. Previous meta-analyses and reviews revealed that cheese intake is associated with a decline in blood lipid levels, arterial stiffness measured by pulse wave velocity, and stroke risk [19,20].

In light of these previous studies, longitudinal studies on the effects of hypertension on cognitive functions and brain morphology during aging have not clarified the following issues: (a) the relationships between BP and longitudinal changes in brain microstructure as measured by DTI and NODDI; (b) the voxel-by-voxel relationships between BP and longitudinal changes in imaging values after correction for baseline values using a larger sample; (c) elucidation of decreased cognitive functions (if any) in the hypertension after adjustment of cofounders in the modern life; and (d) the impacts of the dietary intake that is associated with diastolic BP changes on each outcome measure. This study aims to clarify the abovementioned issues. Clarifying these issues is important because, in modern life, the effective treatment against stroke has evolved and some of the associations between lifestyle-related factors (body mass index) and the risk of dementia have been reversed compared with what has been previously reported [21,22].

To address these issues, we analyze data from the UK Biobank, which includes a wide range of longitudinal cognitive and imaging measures from a large sample of middle-aged to elderly adults. To measure the microstructural properties of the brain, such as FA, MD, ICVF, and ISOVF, we use DTI and NODDI [23]. Combining these measures with regional gray matter volume (rGMV) and regional white matter volume (rWMV) as estimated by voxel-based morphometry (VBM) [24] allows for a comprehensive evaluation of longitudinal changes in brain structural properties during aging. Further, the current study employs biological parametric mapping (BPM), which allows for voxel-by-voxel (meaning brain regions are separated into small voxels, which allow for the evaluation of precise regional changes of the brain) multiple regression analyses adjusted for confounding imaging variables, such as baseline measurements and tissue concentrations at each voxel. We primarily focused on diastolic BP, as it is more robustly associated with dementia risk over time in middle life compared with the systolic BP [3] and is more relevant to the current study’s aims. For the purpose of (d), we chose the amount of cheese intake as an independent measure due to the above-mentioned background.

Identifying the associations between BP and neurocognitive changes in the aging population is of great clinical and social significance given the high prevalence of hypertension and aging population in many countries. However, given the inconsistencies in previous investigations, we tested two opposing hypotheses. Higher BP could be associated with more severe structural and neurocognitive deterioration with age. Specifically, higher BP would be associated with the acceleration of structural changes in aging, including more rapid declines in rGMV, rWMV, FA, and ICVF, and greater increases in MD, AD, RD, and ISOVF [11,12]. This first hypothesis is based on a series of longitudinal and cross-sectional studies showing that higher BP in later life is associated with poorer cognitive outcomes and on previous cross sectional imaging studies generally showing associations between higher BP and greater changes in structural metrics. Conversely, it is possible that hypertension is associated with subsequent changes that are opposite to changes seen in aging and dementia, as multiple studies have found that higher BP is associated with a reduced risk of dementia, while theoretical considerations also posit that higher BP have an aspect of self-protection of the brain that suffers from hypoperfusion [25].

## 2. Methods

**Figure 1:**
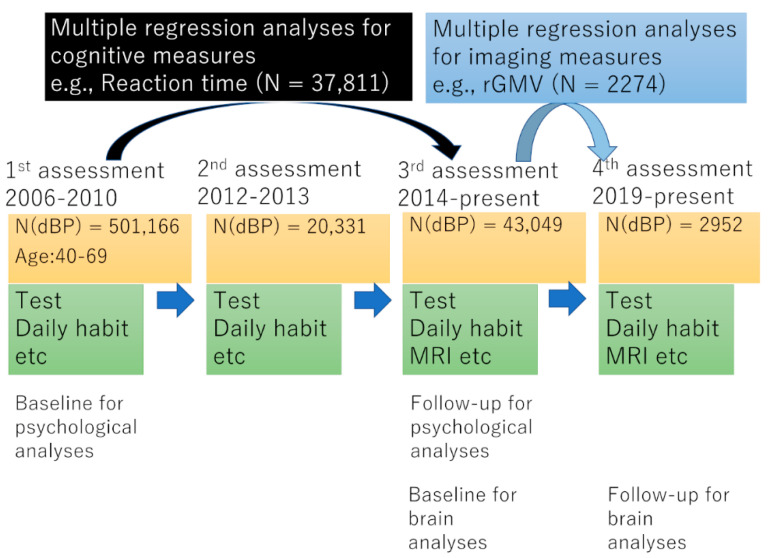
The scheme of study flow and assessment in the UK Biobank and the analyses of the present study. N(dBP) represents numbers of subjects whose data of diastolic blood pressure (BP) are available in each assessment occasion.

**Participants.** The present study data were obtained from a prospective cohort study of middle-aged individuals in the United Kingdom (for procedures, refer to https://www.ukbiobank.ac.uk/media/gnkeyh2q/study-rationale.pdf, accessed on 5 December 2021). Approval for the current analyses was obtained from the North-West Multi-center Research Ethics Committee, and written informed consent was obtained from each participant. Briefly, participants visited 1 of 22 assessment centers throughout the UK, and baseline data were obtained from 502,505 participants. Our study included data from the cohort that completed the first assessment visit (2006–2010), the third assessment visit, during which the first imaging dataset (baseline) was collected (2014 to the present), and from the fourth assessment visit, during which follow-up imaging data were collected (2019 to the present). The following analyses were conducted from the data of subjects whose all dependent and independent data were available in each analysis. The scheme of these four assessments in the UK Biobank and analyses in the present study is shown in Figure 1.

All the experimental procedures involving human participants followed the ethical standards of the institutional and/or national research committee and the 1964 Helsinki Declaration and its later amendments or comparable ethical standards. The descriptions in this subsection are largely reproduced from our previous study using the same methods [26].

**Blood pressure measurements.** Diastolic blood pressure was measured using a digital BP monitor (Omron) or manual sphygmomanometer when the digital monitor was not available (data-field IDs: 94, 4079). One or two readings were taken and the average recorded as described in a previous study [27]. Diastolic BP was measured at all the four assessment occasions (Figure 1).

**Sociodemographic and lifestyle measurements used as covariates.** Self-reported gender data were used. From the database, the neighborhood-level socioeconomic status at recruitment (cov1), education level at recruitment (cov2), household income (cov3), current employment status (cov4), metabolic equivalent of task hours (MET) (cov5), number in household (cov6), body mass index (BMI, cov7), self-reported health status (cov8), sleep duration (cov9), current alcohol drinking status (cov10), current tobacco smoking level (cov11), ethnicity (white or not) (cov12), depression score (cov13), and use of antihyperintensive medication (Yes or No, cov14) (for additional details, refer to Supplemental Methods). The descriptions in this subsection are largely reproduced from our previous study using the same methods [26].

**Cognitive measures.** Cognitive measures were administered at all visits. Briefly, tests were administered through a computerized touchscreen interface at each assessment center. In the current study, we used the data for fluid intelligence (non-verbal reasoning), reaction time (symbol matching), visuospatial memory (pair matching), and depressive symptoms measured by the 4-item Patient Health Questionnaire-4 (PHQ-4) [28]. For additional details, please refer to Supplemental Methods. The descriptions in this subsection are largely reproduced from our previous study using the same methods [26].

**Structural magnetic resonance imaging (MRI) acquisition and preprocessing for volumetric analyses.** MRI data were obtained during the third and fourth assessment visits at three imaging centers equipped with identical scanners (Siemens Skyra 3T running VD13A SP4 with a Siemens 32-channel RF receive head coil, Munich, Germany).

T1-weighted structural images (data id = 25,252) were used for the VBM analyses. Images were first segmented, and then the segmented GM and WM were normalized using the diffeomorphic anatomical registration through the exponentiated lie algebra (DARTEL) procedure. Normalized images were modulated and smoothed using an 8 mm full width at half-maximum (FWHM) Gaussian kernel to yield rGMV and rWMV maps. For further details, see Supplemental Methods.

For microstructural analyses, we used metrics derived from DTI and NODDI [23] (data id = 25,250). We then derived the following measures from DTI: (a) MD, which reflects the extent of water molecule diffusion regardless of direction; (b) axial diffusivity (AD), which reflects water molecule diffusion parallel to WM tract direction within a voxel of interest; (c) radial diffusivity (RD), which reflects the magnitude of water diffusion perpendicular to the tract direction; and (d) fractional anisotropy, which reflects the anisotropy of water diffusion. Similarly, NODDI was used to derive the following measures: (A) the ICVF, which reflects neurite compartment density as verified by histology in animal experiments [29]; (B) ISOVF, which reflects extracellular free water diffusion as well as interstitial and cerebrospinal fluid (CSF) volumes; and (C) orientation dispersion (OD) index, which reflects the spread of fibers within an intracellular compartment.

Briefly, diffusion images were constructed from MD and FA measures and normalized using a previously validated modified DARTEL procedure [30] that accounts the FA signal distribution within WM areas (to align images and tracts) as well as regional GM density (rGMD), regional WM density (rWMD), and regional cerebrospinal fluid density (rCSFD). All normalized DT and NODD images were masked using a WM tissue probability (>0.99) map to limit analyses to white matter areas, and then smoothed (6 mm FWHM). For more details on these procedures, please refer to Supplemental Methods.

**Whole-brain imaging data analysis.** The Biological Parametric Mapping tool (BPM; www.fmri.wfubmc.edu, accessed on 25 August 2019) [31], an extension software suite for Statistical Parametric Mapping (SPM; Wellcome Trust Centre for Human Neuroimaging, London, UK; available at: http://www.fil.ion.ucl.ac.uk/spm, accessed on 7 June 2022), was used for all statistical analysis of imaging data. Longitudinal whole-brain multiple regression analyses were used to identify associations between diastolic BP and brain imaging parameters (rGMV, rWMV, DTI metrics, and NODDI metrics) from the third assessment visit as baseline values and the fourth visit as follow-up values (as imaging data were available only from the third and fourth assessment visits).

In rGMV and rWMV analyses, the independent variables were sex, age at the third assessment visit, the interval (in days) between the third and fourth assessment visits, cov1–cov14 values at the third assessment visit (except for cov1 and cov2, which are stable values obtained at recruitment), head size ratio at the third assessment visit (calculated using UK Biobank output, in UK Biobank; head size normalization was performed using T1-based “head size scaling factor,” data id = 25,000, which is a scaling factor estimated when transforming from native to standard space), BMI at the third assessment visit, and imaging measurements at each voxel during the third assessment visit. Pre-imaging measurement effects were corrected on a voxel-by-voxel basis using BPM. The dependent variables at each voxel were the follow-up imaging metrics from the fourth assessment visit. The *p*- and t-values were the same when baseline measurements were included as independent variables regardless of whether the outcome measurement at follow-up assessment or the differences between baseline and follow-up outcome measurements were used as dependent variables. The results of longitudinal analyses were thus interpreted as the associations between diastolic BP at baseline and the changes in each imaging parameter between baseline and follow-up. For DTI and NODDI images, analyses were performed in a similar manner as rGMV and rWMV analyses except that rWMD and rCSFD images of the third and fourth assessment visit were included as covariate images to correct the effects of WM and CSF probabilities.

For rGMV and rWMV analyses, only voxels with signal intensity > 0.10 for all subjects in whole-brain analyses were included. Fractional anisotropy image analysis was limited to the areas of white matter tissue probability > 0.99 and other DTI and NODDI analyses were limited to areas of white matter tissue probability > 0.99 (see Supplemental Methods for a description of mask creation procedures).

Statistical tests were corrected for multiple comparisons using the false discovery rate (FDR) approach [32]. Areas that surpassed the extent threshold [33] based on a cluster-determining threshold *p* < 0.05 were corrected for FDR, as described in our previous study [34].

**Psychological and non-whole brain imaging data analyses.** Psychological and non-whole-brain imaging data were analyzed using Predictive Analysis Software, version 22.0.0 (SPSS Inc., Chicago, IL, USA; 2010). Multiple regression analyses were used to investigate the associations between diastolic BP at the first assessment visit and changes in cognitive variables from the first to the third assessment visit with correction for confounding variables. The change in each measured variable from the first to the third assessment visit was used because the second assessment contained less data for psychological analyses than the third. The dependent variables included in each multiple regression model were changes in (A) fluid intelligence, (B) reaction time, (C) visuospatial memory, and (D) depressive symptoms from the first to the third assessment visit, and the independent variables were sex, age at the first assessment visit, interval (in days) between the first and third assessment visits, the number of times subjects underwent tests for this project at the time of the third assessment visit, cov1–cov14 values on the first assessment visit, and diastolic BP on the first assessment visit.

For non-whole-brain imaging analyses, we used the mean or total imaging variable of each imaging measure (rGMV, rWMV, FA, MD, AD, RD, ICVF, ISOVF, rWMD, and rCSFD) in the third and fourth assessment visits, and other covariates were the same as those of whole-brain imaging analyses.

For psychological and non-whole-brain imaging analyses, the results with a threshold *p* < 0.05 corrected for FDR using the two-stage sharpened method [35] were considered statistically significant. This correction was applied to the results of the four analyses described above and nine analyses of the mean and total imaging variables described above.

**Analyses of the nutritional modulation of diastolic BP.** The methods of collecting dietary data in the UK Biobank were previously described [36]. Participants answered questions about the frequency of cheese consumption along with other foods on the touchscreen at each assessment visit. In the conversion of dietary processes, we followed a representative study using this data [36]. The subjects were divided into four groups according to their food intake (Level 1 (≤once/wk), Level 2 (once/wk), Level 3 (2–4 times/wk), and Level 4 (≧5 times/wk)). The details are presented in the Supplemental Methods.

For statistical analyses, we conducted non-whole-brain analyses of covariance (ANCOVAs). In each analysis, the dependent and independent measures were the same as those described in the above-mentioned main multiple regression analyses that evaluate the effects of diastolic BP, except that the cheese intake level was added as a fixed factor of the independent variable. In addition, we evaluated the impacts of cheese intake level at baseline on the change in diastolic BP from the first assessment to the third assessment. Corrections of multiple comparisons were conducted using the FDR of the two-stage sharpened method [35], among which were 14 ANCOVAs for the assessment of cheese intake level.

## 3. Results

### 3.1. Basic Baseline Data

Baseline psychological data for all participants acquired at the first assessment are summarized in Supplemental Table S1. Simple correlation coefficients for the associations between diastolic BP and the psychological variables used as covariates in subsequent multiple regression analyses were all <0.30, so there was no potential for multicollinearity.

### 3.2. Longitudinal Psychological Analyses

**Figure 2:**
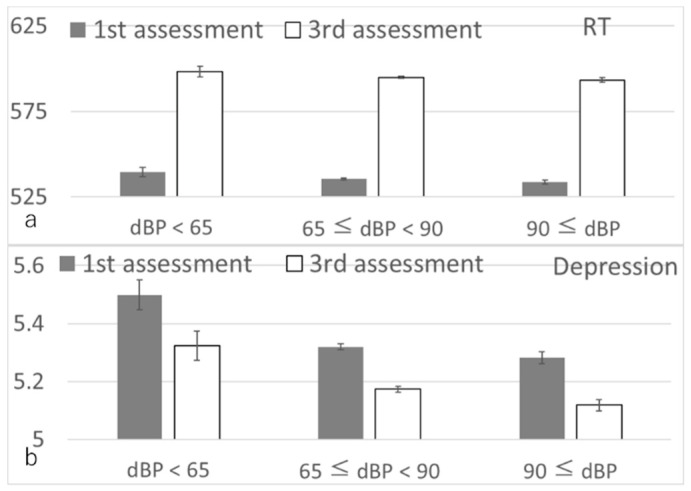
Associations of baseline diastolic BP with subsequent changes in cognitive measures. Bars represent raw unadjusted pre- and post-test values and error bars represent the standard error of the mean. (**a**) Reaction time (RT). (**b**) Depression score. Multiple regression analyses adjusting for confounding variables revealed that greater diastolic BP was associated with a smaller increase in reaction time (smaller decline in cognitive speed) and reduce depressive tendency after adjusting for potential confounding variables, including baseline outcome measures (*p* < 0.05, corrected for FDR).

Baseline and follow-up values for longitudinal psychological analyses were acquired during the first and third assessment visits, respectively. Mean age at first assessment was 56.5 years (standard deviation (SD): 8.0, range: 37–73) and the mean interval between visits was 3278.7 days (SD: 643.6, range: 1400–5055 days). Baseline characteristics of participants included in imaging analyses are provided in Supplemental Table S2. After correcting for confounding variables, multiple regression revealed a small, but significant, association between greater diastolic BP at the first assessment visit and smaller relative increase in reaction time and depression score (i.e., age-corrected relative improvements), but no associations with changes in fluid intelligence and visuospatial task performance (Figure 2, Table 1).

### 3.3. Longitudinal Brain Imaging Analysis

Mean age at the third assessment (baseline image acquisition) was 61.9 years (SD: 7.2, range 46–79), mean diastolic BP was 78.2 (SD: 10.1, range: 49.5–124), and the mean interval between the third and fourth assessment visit (follow-up image acquisition) was 851.6 days (SD: 193.4, range: 366–2457 days) for the 2274 participants included in the rGMV and rWMV analyses.

**Figure 3:**
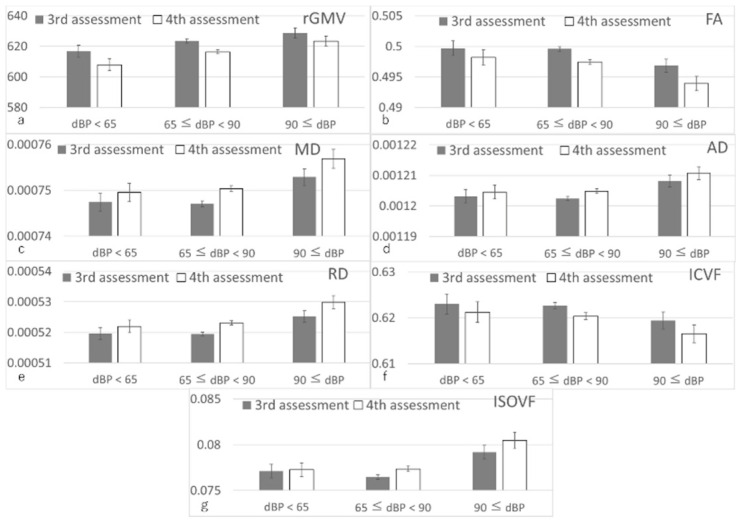
Associations of diastolic BP with subsequent changes in total, mean, or regional imaging measures. Error bars represent the standard error of the mean. (**a**) Total grey matter volume (GMV). (**b**–**g**) Mean white matter metrics: (**b**) functional anisotropy (FA), (**c**) mean diffusivity (MD), (**d**) axial diffusivity (AD), (**e**) radial diffusivity (RD), (**f**) intracellular volume fraction (ICVF), and (**g**) isotropic volume fraction (ISOVF). Multiple regression analyses adjusting for confounding variables revealed that a greater diastolic BP was associated with a subsequent relative increase in rGMV, greater decline in FA, greater increases in MD, AD, and RD, greater decline in ICVF, and greater increase in ISOVF (*p* < 0.05, corrected for FDR).

Non-whole-brain multiple regression analyses of total and regional GMV and WMV revealed significant positive associations between greater diastolic BP at the third assessment and changes in rGMV from the third to the fourth assessment, but not between greater diastolic BP at baseline and changes in rWMV from the third to four assessments (Figure 3). For statistical values, see Table 2.

**Figure 4:**
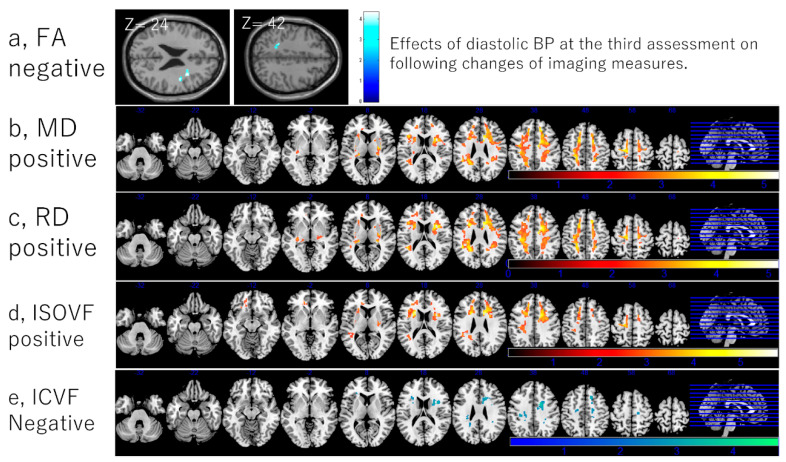
Associations between diastolic BP and subsequent longitudinal changes in brain imaging measures. Associations between a greater diastolic BP and a (**a**) greater FA decline, (**b**) greater MD increase, (**c**) greater RD increase, (**d**) greater ICVF decline, and (**e**) greater ISOVF increase. Results are shown with a threshold *p* < 0.05 and corrections for multiple comparisons in cluster size tests with a voxel-level cluster-determining threshold *p* < 0.05 (corrected for FDR). The color represents the strength of the T-value. (**a**) The findings are overlaid on a “single-subject T1” SPM5 image. (**b**–**e**) Areas of significant associations are overlaid on a “ch2bet” image using MRIcron (https://www.nitrc.org/projects/mricron, accessed on 5 December 2021) and in slices (from the left) of z = −32, −22, −12, −2, 8, 18, 28 38, 48, 58, and 68.

The whole-brain multiple regression analyses of the VBM metrics revealed no significant associations between diastolic BP at the third assessment and changes in rGMV and rWMV from the third to fourth assessments. However, there was a positive trend between diastolic BP at the third assessment and rGMV change in proximity to the right precuneus and postcentral gyrus (x, y, z = 18, −42, 63; (t = 4.09 and *p* = 0.119 corrected for FDR at the peak voxel); 4509 mm^3^ at the threshold of uncorrected *p* < 0.001) (Figure 4).

Non-whole-brain multiple regression analyses using mean diffusion measures revealed that a greater diastolic BP at the third assessment was significantly associated with a greater relative decline in mean white matter FA, greater relative increases in mean white matter MD, AD, and RD, a greater relative decline in mean white matter ICVF, and a greater relative increase in mean white matter ISOVF (Figure 3, see Table 2 for statistical values).

Whole-brain voxel-based multiple regression analyses revealed significant associations of a greater diastolic BP at the third assessment with a greater relative FA decline in several widespread clusters located mainly around the bilateral corona radiata, superior longitudinal fasciculus, and internal and external capsules. These analyses also revealed similar spatial correlation patterns for greater diastolic BP at the third assessment with a larger relative MD, RD, and ISOVF increases. In these analyses, large bilateral clusters were located mainly in dorsal white matter areas. Clusters were usually spread primarily around the bilateral corona radiata, superior longitudinal fasciculus, internal and external capsules, and small peripheral regions of the corpus callosum. No significant correlations were observed in AD analyses; however, at a threshold of *p* < 0.1 (corrected for FDR), sporadic clusters of positive correlations were observed in areas similar to those identified by FA analyses, consistent with non-whole-brain regression analyses using mean AD areas (Figure 4; for statistical values, see Supplemental Tables S3–S6).

Three clusters of significant associations were also found between a greater diastolic BP at the third assessment and greater ICVF declines among the same areas as FA and AD associations, but the spatial extents were more confined. These significant clusters were found in the right superior corona radiate, right superior longitudinal fasciculus, and the posterior white matter (Figure 4). No significant correlations were found in OD analyses.

Supplemental comparisons of statistical results of the effects of diastolic BP with and without the inclusion of major relevant comorbidities as covariates.

As described in the Introduction Section, we primarily focused on diastolic BP, as it is more robustly associated with dementia risk over time in middle life than systolic BP [3] and is more relevant to the current study’s aims. However, other studies suggest that systemic BP is slightly more relevant, or at least equally important, for BP association with stroke [37]. Therefore, we added supplementary analyses that replaced the covariates of diastolic BP with those of systolic BP in the same dataset. In imaging analyses, we used the total or mean imaging values (total rGMV and mean FA) for independent variables of imaging measures and changes in the total or mean imaging values for dependent variables, as was the case in Table 2. Statistical values for comparisons are provided in Supplemental Table S7.

The results show that, for most of the results, significance (at uncorrected level) and insignificance did not differ between the results of diastolic BP and those of systolic BP (significant: changes in reaction time, rGMV, MD, AD, RD, and ICVF; insignificant: changes in fluid intelligence, visuospatial memory, rWMV, ISOVF, and PD). However, for the changes in depressive symptoms, the diastolic BP result was significant (*p* = 0.010) and that of systolic BP was slightly insignificant (*p* = 0.057), and for the changes in FA, the diastolic BP result was significant (*p* = 0.004) and that of systolic BP was insignificant (*p* = 0.207). However, with respect to the results of FA and depressive symptoms, the analyses of the systolic BP results showed the same trend as the analyses of the diastolic BP results. Therefore, the systolic BP results resembled those of diastolic BP, and these two changes in the status of significance did not affect our discussions.

**Evaluation of the impacts of the adjustment of major relevant comorbidities on the associations between diastolic BP and longitudinal changes in outcome measures**.

We conducted sensitivity analyses using psychological measures and total or mean imaging measures as outcome variables in which the diagnosis of stroke, hyperlipidity, diabetes, and myocardial infarction at baseline were included as covariates. The details of the methods are provided in Supplemental Methods, and statistical values are provided in Supplemental Table S8. Overall, the adjustment of the existence of these major comorbidities did not affect the significance and insignificance of the results.

**Associations between baselinecheese intake and longitudinal changes indiastolic BPandpsychological and morphometric or diffusivity parameters revealed by multiple regression analyses**.

**Figure 5:**
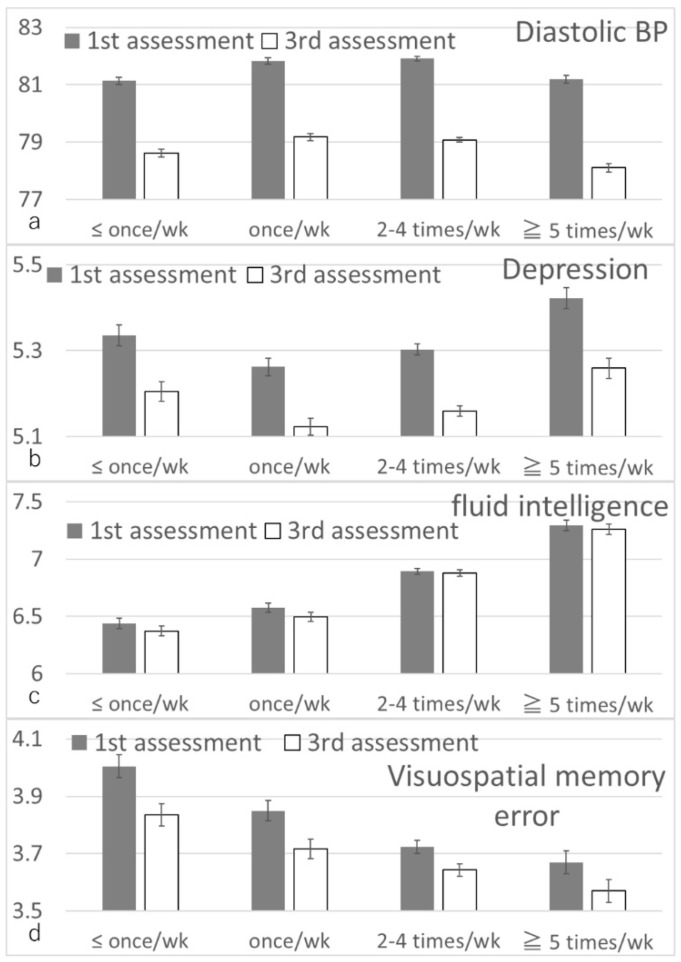
Associations of baseline cheese intake with subsequent changes in diastolic BP and cognitive measures. Bars represent raw unadjusted pre- and post-test values and error bars represent the standard error of the mean. (**a**) Diastolic BP. (**b**) Depression score. (**c**) Fluid intelligence. (**d**) Visuospatial memory errors. Analyses of covariance (ANCOVAs) adjusting for confounding variables including pre-test values showed that a greater cheese intake was associated with a greater diastolic BP decline, greater depression score increase, relatively increased fluid intelligence, and relatively decreased visuospatial memory errors (*p* < 0.05, corrected for FDR).

After correcting for confounding variables and corrections for multiple comparisons, ANCOVAs revealed associations between cheese intake level at the first assessment and the changes in the following variables between the first and third assessments: longitudinal diastolic BP, fluid intelligence, visuospatial memory performance, and depressive tendencies (Figure 5). Post hoc analyses revealed that, in each measure, compared with the groups with a lower cheese intake level, the groups with a greater cheese intake level at baseline were associated with a greater longitudinal diastolic BP decline, greater longitudinal fluid intelligence relative increase (retention), greater visuospatial memory error decline (performance relative increase), and greater depression relative increase (less decline).

Significant associations were not observed between cheese intake level at the third assessment and the longitudinal mean or total imaging parameters.

The mean changes in each group and statistical values are presented in Table 3.

## 4. Discussion

We conducted the first systematic large-scale investigation of the relationships between diastolic BP and subsequent changes in DTI and NODDI measures as well as rGMV and rWMV using the voxel-by-voxel method. A baseline higher diastolic BP was associated with (i) a greater retention of total GMV, (ii) a greater mean reduction in white matter FA and greater regional white matter FA reductions within several dorsal tracts, (iii) larger mean white matter MD, RD, AD, and ISOVF increases and regional MD, RD, and ISOVF increases in widespread white matter tracts, (iv) and a greater decline in mean white matter ICVF. We also found that higher diastolic BP was not significantly associated with worse cognitive outcome measures despite the large sample size and correction for a wide range of confounders. Rather, higher diastolic BP was associated with a slight, but significant, relative increase in cognitive speed and slight reduction in the depression score. These results were mostly not affected by the correctional effects of the existence of major diseases relevant to high BP, such as diabetes, myocardial infarction, stroke, and hyperlipidemia at baseline. The use of systolic BP as a predicter mostly did not induce insignificant results, and the same tendencies were observed, even when the significant results were lost. Furthermore, a greater cheese intake level at baseline was associated with a greater longitudinal diastolic BP decline, greater longitudinal fluid intelligence relative increase (retention), greater visuospatial memory error decline (performance relative increase), and greater depression relative increase (less decline). These findings further underscore the importance of diastolic BP as a factor associated with the multiple brain mechanisms of the aging brain and suggest that the complex neurocognitive consequences of higher BP must be considered when controlling BP. Cheese intake may be considered a nonpharmaceutical candidate that affects diastolic BP, and future intervention studies must confirm these notions.

We found many inconsistencies with both of our original hypotheses. The changes in rGMV and reaction time were consistent with our first hypothesis and in direct opposition to previous findings on the effects of aging. While aging was associated with a decline in rGMV and slower reaction time (cognitive speed), higher BP was associated with a greater retention of rGMV and a slight improvement in reaction time (although the effect size was very small). Further, higher BP was associated with a slight decline in depressive symptoms. On the other hand, the results of diffusion imaging measures were consistent with the second hypothesis, as higher BP was associated with changes similar to those associated with aging. Both aging and high BP were associated with decline in mean FA, increase in mean MD, decline in ICVF, and increase in ISOVF.

These neuroimaging changes generally suggest associations of higher diastolic BP with the retention of various gray matter components, but damage to various white matter structures. For example, a greater rGMV retention suggests a more efficient preservation of neuronal and glial cell number and size, greater synaptic density, and larger neurites in gray matter [38]. On the other hand, a decline in FA suggests reduced myelination, axonal membrane thickness, and axonal diameter [39]. Uniformly increased MD, AD, and RD may reflect a decline in tissue structures that normally prevent the free diffusion of fluid and ensuing changes in fluid volume [39]. Similarly, a decline in ICVF may reflect a parallel decline in density of neurites (axon and dendrites) [40], while an increase in ISOVF is suggestive of a greater extracellular free water diffusion and interstitial fluid volume [29]. These findings are consistent with the associations of higher BP with lower FA, greater MD, lower ICVF, and higher ISOVF using the cross-sectional data of the UK Biobank and also suggest the common associations of BP with white matter microstructural properties in cross-sectional and longitudinal associations. The mechanisms underlying the observed associations of higher BP with a greater GMV retention and changes in diffusion measures during aging are uncertain, but again suggest differential effects on WM and GM. Although the potential mechanisms of this differential effect were speculative, they are provided in the Supplemental Discussion.

In contrast to volumetric measures, we found no significant associations between higher baseline diastolic BP and subsequent negative cognitive outcomes, despite the large sample size (N > 10,000). In fact, we actually found weak, but significant, associations between higher baseline diastolic BP and a better outcome (slower RT decline and reduced depressive tendencies). This is in clear contrast to previous findings of cognitive declines in aging individuals with high BP [6]. However, these studies were generally of limited sample size, which limits covariate correction. In this study, the removal of covariates other than age, sex, and interval led to tendencies or even significant positive associations between greater BP at baseline and the retention of fluid intelligence (beta = 0.013, *p* = 0.084) and visuospatial memory performance (beta = −0.010, *p* = 0.005). These results further imply that correction for a wide range of potential confounders cannot account for the discrepancies with previous studies. Recent advances in treatments for BP-related diseases, such as stroke, may explain these discrepancies, as such treatments may eliminate subpopulations contributing to the negative impacts of higher BP on cognition reported in older datasets. Similarly, while a higher BMI is traditionally associated with subsequent dementia onset [41], recent megasample studies found the opposite patterns [21,22]. These results may be consistent with the view that higher BP have an aspect of self-protection of the brain that suffers from hypoperfusion [25], or the view that that low BP in the late life is the pre-clinical characteristic of dementia [4]. However, ultimately well-designed intervention studies are required to confirm these views.

The present associations between a greater cheese intake and longitudinal decline in diastolic BP, relative increase in fluid intelligence, visuospatial memory, and depressive tendency are mostly consistent with previous interventional, longitudinal, or cross-sectional findings [16,17,19,20,42], and we provide possible speculative mechanisms for these associations. As described in the Methods Section, cross-sectional and interventional studies demonstrated the associations of cheese intake in general or cheese of certain types with lower or decline in BP, and meta-analyses demonstrated the associations of cheese intake with measures that are closely relevant to BP, such as stroke risk, arterial stiffness, and blood lipid [16,17,19,20,42]. In addition, using the same UK Biobank data, a greater cheese intake was shown to be associated with a greater longitudinal retention (less decline) in fluid intelligence [43]. We further demonstrated that greater cheese intake is associated with a greater retention of visuospatial memory performance, whereas previous studies failed to show this, possibly due to limited sample size [44]. Finally, while only a few studies have demonstrated the cross-sectional association of a greater cheese intake with greater depression levels [45], we showed that a greater cheese intake is associated with longitudinal change toward a greater depressive tendency. The mechanisms of these associations are unclear and we can only speculate. Peptides with ACE-inhibiting or BP-lowering activity have been identified in many cheeses [18], and this is an obvious candidate for the mechanism of the present observations between cheese intake and longitudinal BP changes. However, the small, but significant, effects of calcium on lower BP may also be relevant [46]. The depressive relative increase in the group with a greater cheese intake may support the fact that greater cheese intake habits are associated with a decline in BP and that higher BP is associated with decreased depressive tendencies. The effects of cheese on cognitive functions may not be due to the effects of BP, given that diastolic BP itself was not associated with subsequent cognitive function changes. However, this is an observational longitudinal study, and causality is entangled as cheese includes various nutrients. These speculations cannot be determined from the present study, and future studies need to investigate this issue. Furthermore, the cheese intake habit was not associated with imaging indices, despite their association with the change in diastolic BP. This may be because the sample size was not large enough for the investigation of these associations, as the effect of cheese intake on diastolic BP was small, and then the associations between diastolic BP and longitudinal changes in brain structures were also weak. Future studies will need to re-evaluate how cheese intake induces changes in brain structures, whether the associations of cheese intake and memory retention and depressive increase are replicated in longitudinal and intervention studies.

This is a longitudinal study of prospective analyses. Although multiple ways of associating BP with outcomes exist, we concentrated on the associations between baseline exposure variables and longitudinal outcome changes, as cases of our previous studies of this kind exist [47,48]. This is because, as described previously [47], to indicate causality at certain levels with the analyses in a prospective longitudinal cohort study, it is important that certain variables measured in the baseline predict (or precede) subsequent changes in outcome variables. It is the gold standard procedure for prospective longitudinal studies.

This study has a few limitations. First, although the study design was prospective and longitudinal, there were no interventions, so it is difficult to completely exclude reverse causations. As described in the Introduction, some studies suggest that low BP is a symptom (not a cause) of pre-clinical dementia [4], while a Mendelian randomization study suggested a causal effect of lower BP on dementia risk [5]. The neurological processes of Alzheimer’s disease start 20 years before onset [49], and disentangling causes from outcomes over this period is difficult. Further, many factors can impact BP, and factors driving higher or lower BP, rather than BP itself, may contribute to the associations observed in the present study. Future interventional studies (such as lowering BP through medical interventions) may be required to distinguish among these possibilities. In addition, the participants who attended the follow-up assessments may be biased in that they had not deceased and may be relatively cognitively intact enough to attend the experiments, as are the cases of all of these cohort studies in the elderly.

In summary, in this longitudinal study of the middle age to old adults, higher baseline diastolic BP was associated with slight, but significantly better, cognitive outcomes (relative increase in cognitive speed and slight reduction in depression score), rGMV retention, which is opposite to that seen during the aging process, and white matter changes in the same direction as seen in aging. Also a greater cheese intake level at baseline, was associated with a subsequent decline in diastolic BP and a relative subsequent increase in depressive tendency together with better outcomes of cognitive functions.

## Figures and Tables

**Table 1:** Associations between baseline diastolic blood pressure and longitudinal changes in psychological measures revealed by multiple regression analyses.

Dependent Variables	*N*	Standardized Beta	T	*p* (Uncorrected)	*p* (FDR)
Fluid intelligence	12,827	−0.002 (−0.019, 0.014)	−0.283	0.777	0.439
Reaction time	37,811	−0.012 (−0.022, −0.003)	−2.478	0.013	0.019
Visuospatial memory (number of errors)	37,261	−0.004 (−0.012, 0.005)	−0.834	0.404	0.270
Depressive symptoms	38,461	−0.012 (−0.021, −0.003)	−2.589	0.010	0.019

FDR: false discovery rate. Each analysis included sex, age at the first assessment visit, interval (in days) between the first and third assessment visits, the number of times subjects underwent tests for this project at the time of the third assessment visit, cov1–cov14 values (which are described in the Methods Section) on the first assessment visit, and diastolic BP on the first assessment visit.

**Table 2:** Associations between baseline diastolic BP (third assessment) and longitudinal changes in morphometric or diffusivity parameters revealed by multiple regression analyses.

Dependent Variables	N	Standardized Beta	T	*p* (Uncorrected)	*p* (FDR)
rGMV	2274	0.045 (0.002~0.088)	2.067	0.039	0.037
rWMV	2274	−0.022 (−0.065~0.021)	−0.991	0.322	0.237
FA	2240	−0.066 (−0.11~−0.022)	−2.922	0.004	0.015
MD	2240	0.055 (0.012~0.098)	2.502	0.012	0.019
AD	2240	0.044 (0.002~0.087)	2.051	0.040	0.037
RD	2240	0.064 (0.021~0.107)	2.933	0.003	0.015
ICVF	2240	−0.05 (−0.093~−0.008)	−2.306	0.021	0.026
ISOVF	2240	0.043 (−0.001~0.087)	1.929	0.054	0.044
OD	2240	−0.014 (−0.055~0.027)	−0.682	0.496	0.304

rGMV, regional grey matter volume; rWMV, regional white matter volume; FA, fractional anisotropy; MD, mean diffusivity; AD, axial diffusivity; RD, radial diffusivity; ICVF, intracellular volume fraction; ISOVF, isotropic volume fraction; OD, orientation dispersion. Each analysis included sex, age at the third assessment visit, interval (in days) between the third and fourth assessment visits, the head size ratio at the third assessment, cov1–cov14 values (which are described in the Methods Section) on the first assessment visit, and diastolic BP on the third assessment visit.

**Table 3:** Associations between baseline cheese intake and longitudinal changes in diastolic BP and psychological and imaging parameters revealed by multiple regression analyses.

Dependent Variables	*N*	Level 1 (<Once/wk) Mean Change (95%CI)	Level 2 (Once/wk) Mean Change (95%CI) *p* (Level 1 vs. Level 2)	Level 3 (2–4 Times/wk) Mean Change (95%CI) *p* (Level 1 vs. Level 3)	Level 4 (≥5 Times/wk) Mean Change (95%CI) *p* (Level 1 vs. Level 4)	Group Level Difference *p*-Value (Uncorrected, FDR)
Diastolic BP	34,964	−2.676 (−2.895~−2.458)	−2.597 (−2.795~−2.398) 0.594	−2.747 (−2.874~−2.62) 0.589	−3.267 (−3.494~−3.04) 2.63 × 10^−4^	7.28 × 10^−5^ 3.82 × 10^−4^
Fluid intelligence	12,636	−0.15 (−0.218~−0.083) -	−0.151 (−0.212~−0.089) 0.996	−0.003 (−0.042~0.035) 2.25 × 10^−4^	0.09 (0.024~0.155) 7.95 × 10^−7^	1.21 × 10^−8^ 1.27 × 10^−7^
Reaction time	37,185	59.5 (57.2–61.8) -	60.6 (58.5~62.8) 0.467	59.3 (57.9~60.6) 0.899	56.8 (54.4~59.2) 0.114	0.123 0.215
Visuospatial memory (errors)	36,653	−0.026 (−0.098~0.046) -	−0.07 (−0.135~−0.004) 0.377	−0.123 (−0.165~−0.081) 0.024	−0.198 (−0.272~−0.124) 0.001	0.007 0.018
Depressive symptoms	37,814	−0.152 (−0.188~−0.115) -	−0.179 (−0.212~−0.146) 0.265	−0.145 (−0.166~−0.124) 0.770	−0.085 (−0.123~−0.048) 0.013	0.003 0.011
rGMV	2233	−6655 (−7922~−5389) -	−6747 (−7851~−5644) 0.915	−7219 (−7933~−6505) 0.448	−7429 (−8589~−6271) 0.380	0.741 0.750
rWMV	2233	−7298 (−8735~−5860) -	−7787 (−9040~−6534) 0.614	−6586 (−7397~−5776) 0.399	−6867 (−8182~−5551) 0.667	0.441 0.579
FA	2196	−2.1 × 10^−3^ (−2.6 × 10^−3^~−1.6 × 10^−3^) -	−2.6 × 10^−3^ (−3.0 × 10^−3^~−2.1 × 10^−3^) 0.147	−2.0 × 10^−3^ (−2.3 × 10^−3^~−1.7 × 10^−3^) 0.763	−2.5 × 10^−3^ (−2.9 × 10^−3^~−2.0 × 10^−3^) 0.265	0.094 0.197
MD	2196	3.4 × 10^−6^ (2.4 × 10^−6^~4.5 × 10^−6^) -	3.6 × 10^−6^ (2.7 × 10^−6^~4.5 × 10^−6^) 0.828	3.1 × 10^−6^ (2.5 × 10^−6^~3.7 × 10^−6^) 0.612	3.6 × 10^−6^ (2.6 × 10^−6^~4.6 × 10^−6^) 0.818	1 0.75
AD	2196	2.7 × 10^−6^ (1.3 × 10^−6^~4.1 × 10^−6^) -	2.6 × 10^−6^ (1.3 × 10^−6^~3.8 × 10^−6^) 0.909	2.3 × 10^−6^ (1.5 × 10^−6^~3.1 × 10^−6^) 0.641	2.6 × 10^−6^ (1.3 × 10^−6^~4.0 × 10^−6^) 0.976	1 0.75
RD	2196	3.7 × 10^−6^ (2.8 × 10^−6^~4.7 × 10^−6^) -	4.2 × 10^−6^ (3.3 × 10^−6^~5.0 × 10^−6^) 0.481	3.5 × 10^−6^ (3.0 × 10^−6^~4.0 × 10^−6^) 0.690	4.1 × 10^−6^ (3.2 × 10^−6^~4.9 × 10^−6^) 0.600	1 0.750
ICVF	2196	−1.9 × 10^−3^ (−2.6 × 10^−3^~−1.2 × 10^−3^) -	−2.4 × 10^−3^ (−3.0 × 10^−3^~−1.8 × 10^−3^) 0.34	−2.5 × 10^−3^ (−2.9 × 10^−3^~−2.1 × 10^−3^) 0.186	−2.3 × 10^−3^ (−3.0 × 10^−3^~−1.6 × 10^−3^) 0.436	0.620 0.723
ISOVF	2196	1.2 × 10^−3^ (0.5 × 10^−3^~1.9 × 10^−3^) -	1.1 × 10^−3^ (0.5 × 10^−3^~1.8 × 10^−3^) 0.846	0.6 × 10^−3^ (0.2 × 10^−3^~1.0 × 10^−3^) 0.14	1.0 × 10^−3^ (0.3 × 10^−3^~1.7 × 10^−3^) 0.634	0.337 0.506
OD	2196	4.7 × 10^−4^ (1.3 × 10^−4^~8.2 × 10^−4^) -	5.3 × 10^−4^ (2.3 × 10^−4^~8.3 × 10^−4^) 0.773	3.8 × 10^−4^ (1.9 × 10^−4^~5.8 × 10^−4^) 0.679	5.2 × 10^−4^ (2.1 × 10^−4^~8.3 × 10^−4^) 0.821	0.812 0.750

## Data Availability

Researchers can apply to use the UK Biobank resource (https://www.ukbiobank.ac.uk/) and access the data used in the present paper. The batches, scripts, and templates created and used for image preprocessing can be accessed upon request to the first author.

## Supplementary Materials

The following supporting information can be downloaded at: https://www.mdpi.com/article/10.3390/nu14122464/s1, Supplemental Methods, Supplemental Results. Supplemental Discussion. Table S1: Baseline characteristics of UK Biobank participants included in the present project (n = 502,505); Table S2: Characteristics of UK Biobank participants included in the analyses of brain volume analyses (n = 2273); Table S3: Brain regions exhibiting significant negative correlations between diastolic BP and FA changes in longitudinal analyses; Table S4: Brain regions exhibiting significant positive correlations between diastolic BP and MD changes in longitudinal analyses; Table S5: Brain regions that exhibited significant negative correlations between diastolic BP and ICVF changes in longitudinal analyses; Table S6: Brain regions exhibiting significant positive correlations between diastolic BP and ISOVF changes in longitudinal analyses; Table S7: Comparisons of statistical results of the effects of diastolic BP with those of systolic BP; Table S8: Comparisons of statistical results of the effects of diastolic BP with and without the inclusion of major relevant comorbidities as covariates. (See [9,25,26,28,30,34,50,51,52,53,54,55,56,57,58,59,60,61,62,63]).

## Transparency

The guarantors affirm that the manuscript is an honest, accurate, and transparent account of the study, that no important aspects of the study have been omitted, and that any discrepancies from the study as planned (and, if relevant, registered) have been explained.
