# Supplementary material for: Effects of Diastolic Blood Pressure on Brain Structures and Cognitive Functions in Middle and Old Ages: Longitudinal Analyses
Source: Nutrients. 2022 Jun 14;14(12):2464. doi: 10.3390/nu14122464 (PMC9229545; doi:10.3390/nu14122464)
Supplement: Supplementary file 1 [file nutrients-14-02464-s001.zip › nutrients-1710539-supplementary.pdf]

Supplementary online material

Supplemental Methods.

Additional information on subjects

Adults aged 40–69 years registered with the National Health Service (NHS) and living within 25 miles of an evaluation site were invited to take part in the UK Biobank by email. No exclusion criteria were applied for this recruitment.

Details on sociodemographic and lifestyle measures

The following variables were calculated and used in this study. The descriptions in this subsection is largely reproduced from our previous study using the same methods [1].

(cov1) Neighborhood-level socioeconomic status was measured by the Townsend index of material deprivation [2] (via UK Biobank Field ID: 189). Status was calculated based on the home postcode of the subject and represents a composite index of four postcode-level socioeconomic status variables: household overcrowding, unemployment, non-home ownership, and non-car ownership). A higher score implies a lower socioeconomic status. This value was obtained at recruitment and used for all analyses.

(cov2) The education level was based on self-reported data (via UK Biobank Field ID: 6138). Education level categories of participant choices were transformed into numerical values as previously described [3]: “College or University degree” = 20 years; “A levels/AS levels or equivalent” = 13 years; “O levels/GCSEs or equivalent” = 10 years; “CSEs or equivalent” = 10 years; “NVQ or HND or HNC or equivalent“ = 19 years; “Other professional qualifications e.g.,: nursing, teaching” = 15 years; “None of the above” = 7 years; “Prefer not to answer” = missing. This value was obtained at

recruitment and used for all analyses.

(cov3) The household income was the self-reported total income (before taxes) received by the subject's household (via UK Biobank Field ID: 738). The available choices were <£18,000, £18,000 to £30,999, £100,000, £31,000 to £51,999, £52,000 to £100,000, >£100,000, do not know, and prefer not to answer. We converted these choices into ordinal variables of 1–5 (>£100,000 = 5) [4] after excluding answers of do not know and prefer not to answer, as done elsewhere.

(cov4) Current employment status was used to describe the occupation information of the participants (UK Biobank Field ID: 6142). The responses to the variable were: "In paid employment or self-employed," "Retired," "Looking after home and/or family," "Unable to work because of sickness or disability," "Unemployed," "Doing unpaid or voluntary work," "Full or part-time student," and "None of the above." Multiple responses were allowed. Responses were classified as either "In paid employment or self-employed" or not.

(cov5) Physical activity level was calculated from the recorded items from the International Physical Activity Questionnaire short form and was converted into a single measure of total physical activity in metabolic equivalent of task hours (MET) (data-field IDs: 864, 874, 884, 894, 904, and 914). For more details, see the previous study [5].

(cov6) Number of people in their household (including institutions such as care homes) (data-field IDs: 709) was self-reported. Answers were assigned one of four variables: 1 (single person), 2 (two people), 3 (three people), and 4 (four or more people) as has been done elsewhere [6].

(cov7) Body weight was measured using Tanita BC418MA scales (data-field IDs:

21002). Height was measured using a Seca height measure (data-field IDs: 50). BMI was calculated from the measured height and weight.

(cov8) Participants were asked about their health status, with possible answers being excellent, good, fair, poor and converted to values of 4, 3, 2, and 1, respectively, before input in statistical analyses (data-field IDs: 2178).

(cov9) Sleep duration was assessed with the item "About how many hours sleep do you get in every 24 h? (Please include naps.)" (data-field IDs: 1160). Responses were coded as integers and used in analyses after counting sleep durations of less than 3 hours into 3 and sleep duration longer than 10 hours into 10.

(cov10) Current alcohol drinking was calculated as previously described [7]. Study participants were asked to describe their current drinking status (never, previous, current, prefer not to say), and after excluding "prefer not to say" (data-field IDs: 20117), the answers were divided into current or currently not (never, or previous) and coded as 1 and 0.

(cov11) Participants were asked about current tobacco smoking status. Possible answers were 1 (No), 2 (Only occasionally), and 3 (Yes, on most or all days) (data-field IDs: 1239). Responses of "prefer not to answer" were excluded.

(cov12) Ethnicity was self-reported, and possible answers were divided into Caucasian or other and coded accordingly (data-field IDs: 21000).

(cov13) Depressive symptoms were measured by the 4-item Patient Health Questionnaire-4 (PHQ-4) [8], which was administered at all four patients visits to assessment centers (data-field IDs: 2050, 2060, 2070 and 2080). This measurement has an area under the curve of 0.79 for its correlation with a depression diagnosis [9]. For other information on the reliability and validity of this measurement technique, see [9].

(cov14) Patients who reported that they were taking a blood pressure medication (via UK Biobank Field IDs 6153) were coded as 1 and others were coded as 0.

Details of cognitive measures

We used data from tests that were administered to a large portion of subjects. Reasoning, or fluid intelligence, was evaluated using 13 verbal numerical logic and reasoning-type questions with a 2-minute time limit and a maximum score of 13 (data-field IDs: 2016). The Cronbach alpha coefficient for each item was 0.62.

Reaction time was measured using a timed symbol matching test. In this test, for each trial, two cards with symbols were shown and participants had to press a button as fast as possible if the two cards had matching symbols. The score for this task was the mean response time across trials containing matching pairs (data-field IDs: 2023). The Cronbach alpha coefficient for this task was 0.85.

Visuospatial memory was measured by the "pairs-matching' task. In this test, participants were asked to memorize the positions of six card pairs, and then match them from memory while making as few errors as possible. Scores on the pairs-matching test are number of errors that participants made and therefore, higher scores reflect poorer cognitive functions (data-field IDs: 399).

Depressive symptoms were measured by the 4-item Patient Health Questionnaire-4 (PHQ-4) [8], as described above.

The descriptions in this subsection is largely reproduced from our previous study using the same methods [1].

Details of structural MRI acquisition and preprocessing for volumetric analyses

For the UK Biobank study cohort, MRI imaging data was obtained for the third and fourth assessment visits. T1 weighted structural images were obtained from 3 imaging centers equipped with identical scanners (Siemens Skyra 3T running VD13A SP4 with a Siemens 32-channel RF receive head coil, Munich, Germany).

Structural images were acquired that contained straight sagittal orientations with a resolution of 1 × 1 × 1 mm and a field of view of 208 × 256 × 256, were taken over a duration of 5 minutes, and that contained 1-mm isotropic resolution generated using a 3-dimensional magnetization-prepared rapid-acquisition gradient echo.

For segmentation processes, outputs from the standard biobank processing pipeline involving FSL were used. Details of MRI protocols and segmentation are provided elsewhere [10,11].

Normalization processes were performed using Statistical Parametric Mapping software (SPM12; Wellcome Department of Cognitive Neurology, London, UK) implemented in Matlab (Mathworks Inc., Natick, MA, USA). First, using a segmentation algorithm implemented in SPM12, T1-weighted structural images of each scan were segmented, resulting in diffeomorphic anatomical registration through exponentiated lie algebra (DARTEL) import images. White matter segment images were generated through the standard biobank processing pipeline using FSL and were co-registered and resliced with DARTEL import images of white matter segments. Using the same parameters, gray matter segment images generated through the standard biobank processing pipeline using FSL were co-registered and resliced.

Using generated images, the DARTEL registration process was performed using SPM12 and both gray matter and white matter segmentation maps generated through the standard biobank processing pipeline using FSL were normalized to the

Montreal Neurological Institute (MNI) space to give images with 3 × 3 × 3 mm^3 voxels. The DARTEL template was then created using imaging data from the 250 baseline experiment images of subjects from the third assessment visit, as well as 250 follow-up images from the fourth assessment visit of different subjects. Next, using the existing template, DARTEL procedures were performed for all images. In addition, we performed a volume change corrections [12]. rGMV and rWMV images were then smoothed by convolving them with an isotropic Gaussian kernel of 8 mm full width at half maximum.

These processes were performed because although we used DARTEL procedures for precise registration processes, the segmentation quality of the standard biobank processing pipeline using FSL was better than that of SPM12's new segmentation method and CAT 12.

Details of diffusion MRI acquisition and preprocessing

We used DTI and NODDI measurements released by the UK Biobank Imaging Study, including non-normalized FA, MD, AD, RD, ISOVF, ICVF, and OD maps. Details of the dataset can be found in the protocol documentation (https://biobank.ctsu.ox.ac.uk/crystal/docs/brain_mri.pdf) and in a previous study [10]. The key elements from these documents are described below.

Diffusion data were acquired using two b-values (b = 1,000 and 2,000 s/mm^2) with a resolution of 2 × 2 × 2 mm and a field of view of 104 × 104 × 72 over a duration of 7 minutes, with a multiband acceleration factor of 3 in which three slices are acquired simultaneously. For each diffusion-weighted shell, 50 distinct diffusion-encoding

directions were acquired covering 100 distinct directions over two b-values. Both diffusion tensor and NODDI models were fit voxel-wise and the image-derived phenotypes of various model outputs extracted from a set of white matter tracts. Tensor fits utilizing the b = 1000 s/mm^2 data were used to produce maps including MD, FA, RD, and AD. The NODDI model was fit using the Accelerated Microstructure Imaging via Convex Optimization tool [13] with outputs including ISOVF, ICVF, and OD.

Preprocessing and analysis of diffusion data were performed using SPM8 implemented in MATLAB. The following method descriptions have been largely reproduced from our previous study [14,15]. Using the b = 0 image, DTI and NODDI images were linearly aligned to the skull-stripped T2 image template of SPM8 to assist with the following procedures. When this registration processes did not proceed as expected, images were manually aligned to the template image.

A previously validated two-step segmentation algorithm for diffusion images and a previously validated DARTEL-based registration process were normalized [15] in a process involving FA signal distribution within white matter areas. All images, including segments of gray matter (regional gray matter density (rGMD) map], white matter (regional white matter density (rWMD) map], cerebrospinal fluid (CSF) (regional CSF density (rCSFD) map] of diffusion images, were normalized. The voxel size of these normalized images was 2 × 2 × 2 mm^3. In these normalization processes, the template for the DARTEL process was created from 125 baseline (3rd assessment visit) images and 125 follow-up (4th assessment visit) images of different subjects.

The details of these procedures, which have also been described in our previous study [15], are as follows. Using the new segmentation algorithm implemented in SPM8, FA images of each individual subject were segmented into six tissues (first new segmentation). The default parameters and tissue probability maps were used for this process, except that affine regularization was performed using the International Consortium for Brain Mapping template for European brains and the sampling distance (approximate distance between sampled points when estimating the model parameters) was 2 mm. We then synthesized the FA image and MD map. In the synthesized image, the area with a WM tissue probability of >0.5 in the abovementioned new segmentation process was the FA image multiplied by −1 (hence, the synthesized image shows very clear contrast between WM and other tissues); the remaining area is the MD map (for details of this procedure, see below). The synthesized image from each individual was then segmented using the new segmentation algorithm implemented in SPM8 with the same parameters as above (second new segmentation). This two-step segmentation process was adopted because the FA image has a relatively clear contrast between GM and WM, as well as between WM and CSF, and the first new segmentation step can segment WM from other tissues. On the other hand, MD map has clear contrast between GM and CSF and the second new segmentation can segment GM. Since the MD map alone lacks clear contrast between WM and GM, we must use a synthesized image (and the two-step segmentation process).

We then performed a DARTEL registration process in SPM8. We used the DARTEL import image of the GM tissue probability map produced in the second new segmentation process as the GM input for the DARTEL process. The WM input for the

DARTEL process was created as follows. First, the raw FA image was multiplied by the WM tissue probability map from the second new segmentation process within areas having WM probabilities of >0.5 (signals from other areas were set to 0). Next, the FA image * WM tissue probability map was co-registered and resliced based on the DARTEL import WM tissue probability image from the second segmentation, which created the DARTEL import image. The DARTEL template was created using imaging data from 125 baseline (3rd assessment visit) experimental images of subjects and 125 follow-up (4th assessment visit) experimental images of different subjects. Next, using the existing template, DARTEL procedures were performed for all images. The parameters for these procedures were changed as follows to improve accuracy. The number of Gauss–Newton iterations performed within each outer iteration was set to 10 and, in each outer iteration, we used 8-fold more timepoints to solve partial differential equations than the default values. The number of cycles used by the full multi-grid matrix solver was set to 8. The number of relaxation iterations performed in each multi-grid cycle was also set to 8. The resultant synthesized images were spatially normalized to MNI space. Using these parameters, the raw FA, raw MD/AD/RD, raw ICVF, raw ISOVF, raw OD, rGMD, rWMD, and rCSFD maps from the abovementioned second new segmentation process were normalized to give images with $1.5 \times 1.5 \times 1.5\ mm^3$ voxels. The FA image * WM tissue probability map was used in DARTEL procedures because it includes different signal intensities within WM tissues and because the normalization procedure can take advantage of the intensity differences to adjust the image to the template from the perspective of the outer edge of the tissue and within the WM tissue. No modulation was performed in the normalization procedure.

We next created averages from the average images of normalized WM segmentation images (rWMD) of 250 images from which the DARTEL template was created, as described above, from the mask image consisting of voxels with a WM signal intensity >0.99. We then applied this mask image to all normalized images derived from DTI and NODDI maps, therefore retaining from normalized images only areas that are highly likely to represent white matter. These images were then smoothed (6 mm full-width half-maximum) and carried through to second-level analyses of FA.

Evaluation of the impacts of the adjustment of major relevant comorbidities on the associations between diastolic BP and longitudinal changes in outcome measures

We evaluated the impacts of the adjustment of major relevant comorbidities on the associations between diastolic BP and longitudinal changes in outcome measures by including the covariates of comorbidities. These covariates of comorbidities include the following dichotomous variables: (1) self-reported doctor diagnosis of diabetes at baseline (UK Biobank data field ID:2443), (2) self-reported doctor diagnosis of stroke or hospital record of stroke at baseline (UK Biobank data field ID:42006,42007), doctor diagnosis of heart attack (UK Biobank data field ID:6150), doctor diagnosis of angina (UK Biobank data field ID:6150), doctor diagnosis of other serious medical condition/disability (UK Biobank data field ID:2473), hyperlipidemia [defined as self-reported high cholesterol at baseline ((UK Biobank data field ID:2473)), as well as diagnoses in HES records (ICD-9 272.0; ICD-10 E78.0) (ICD-9 272/ICD-10 E78) at baseline, as previously reported [16]]. Here, baseline refers to the data of the first

assessment in psychological longitudinal analyses and the data of the third assessment in brain imaging longitudinal analyses.

We conducted supplemental analyses that included these variables of comorbidities in addition to all other covariates in each of the main analyses and compared the analysis results with the analysis results without covariates of comorbidities. In the imaging analyses, we used the total or mean imaging values (total rGMV, mean FA) for independent variables of imaging measures and changes in total or mean imaging values for dependent variables.

Details of the evaluation of cheese intake

Subjects were asked how often they consumed cheese. The options were "never," "less than one a week," "once a week," "2–4 times a week," "5–6 times a week," "once or more daily," "do not know," and "prefer not to answer." As handled previously [9], after removing the participants with answers of "do not know" and "prefer not to answer," we combined the top two and bottom two frequency choices and divided the subjects into four groups <1.0 time per week, 1.0 time per week, 2.0–4.9 times per week, and ≥5.0 times per week. The descriptions in these subsections were mostly reproduced from our previous study [1].

Supplemental Discussion

The mechanisms underlying the observed associations of higher BP with greater GMV retention and changes in diffusion measures

The mechanisms underlying the observed associations of higher BP with greater GMV retention and changes in diffusion measures during aging are uncertain but again suggest differential effects on WM and GM. Higher BP is known to increase the frequency of WM lesions (WMLs) as manifested by hyperintensities on MRI [17], and this association was near significant in the current study ($p = 0.061$, $t = 1.873$ in the multiple regression analysis correcting for the same covariates as in total GMV analyses). WML is mainly caused by impaired perfusion due to hypertensive microangiopathy and amyloid angiopathy, and may also be associated with decreased cerebral blood flow due to impaired venous perfusion. In some cases, decreased cerebral blood flow due to impaired venous perfusion is also involved and vascular damage due to arteriosclerosis is also a cause of WML [18]. Through these direct and indirect mechanisms, higher BP has been associated with WML. According to the results of a study of WML A study comparing MRI and histological manifestations of deep cerebral WMLs noted activation of vascular endothelial cells and microglia, amyloid angiopathy, arteriosclerosis, demyelination, and enlargement of the perivascular lumen, indicating damage caused by hypoxia. and enlargement of perivascular space [18]. Thus, hypoxia associated with chronic hypertension may account for the microstructural changes described here. On the other hand, empirical evidence also suggests that cerebral hypoperfusion triggers hypertension as a protective mechanism [19]. Indeed, higher BP is associated with better cognitive outcome following ischemic stroke [20]. It is possible that these mechanism may be predominant in GM, resulting in better retention of GMV, while the deleterious effects of chronic hypertension predominant in WM. But these notions are speculative and future studies need to investigate this issue.

Supplemental Table S1. Baseline characteristics of UK Biobank participants included in the present project (n = 502,505)

Item	No. (%)	Mean (SD)	Range
Sex			
Female	273,382 (54.4)		
Male	229,122 (45.6)		
Missing	1		
Age, years		56.5 (8.0)	37-73
Missing	1 (0.0)		
Diastolic BP		82.3 (10.2)	32.0-147.5
65>x	17143 (3.4)		
90>x≧65	371476 (73.9)		
x≧90	112547 (22.4)		
Missing	1340 (0.3)		
BMI		27.4(4.8)	12-75
Underweight (18.5≧x)	2,626 (0.5)		
Normal (25≧x>18.5)	162,523 (32.3)		
Overweight (30≧x>25)	212,097 (42.2)		
Obesity (30>x)	122,153 (24.3)		
Missing	3,107 (0.6)		
Average total household income before tax			
Less than £18,000	97,198 (19.3)		
£18,000 to £30,999	108,177 (21.5)		

£31,000 to £5,1999	110,772 (22.0)		
£52,000 to £100,000	86,266 (17.2)		
Greater than £100,000	22,929 (4.6)		
Missing	77,164 (15.4)		
Townsend index of material deprivation		-1.3 (3.1)	-6-11
Missing	624(0.1)		
Employment status			
In paid employment or self-employed	287,149(57.1)		
Not in paid employment or self-employed	212,404(42.3)		
Missing	2,952(0.6)		
Highest education qualification (years)		13.95(5.1)	7-20
College or University degree (20)	161,163(32.1)		
NVQ or HND or HNC or equivalent (19)	32,727(6.5)		
Other professional qualifications, eg. nursing or teaching (15)	25,804(5.1)		
A levels/As levels or equivalent (13)	55,323(11.0)		
O levels/ GCSEs or equivalent (10)	105,197(20.9)		
CSEs or equivalent (10)	26,887(5.4)		
None of the above (7)	85,271(17.0)		
Missing	10,113(2.0)		
Fluid intelligence		6.0(2.2)	0-13
Missing	339,748(67.2)		

Supplemental Table S2. Characteristics of UK Biobank participants included in the analyses of brain volume analyses (n = 2273).

Item	No. (%)	Mean (SD)	Range
Sex			
Female	1135 (49.9)		
Male	1138 (50.1)		
Age, years		61.9 (7.2)	46-79
Diastolic BP		78.2 (10.1)	49.5-124
65>x	203 (8.9)		
90>x≧65	1791 (78.8)		
x≧90	279 (12.3)		
BMI		26.3(4.2)	13-51
Underweight (18.5≧x)	17 (0.7)		
Normal (25≧x>18.5)	944 (41.5)		
Overweight (30≧x>25)	934 (41.1)		
Obesity (30>x)	378 (16.6)		

Supplemental Table S3. Brain regions exhibiting significant negative correlations between diastolic BP and FA changes in longitudinal analyses.

Included large bundles (number of significant voxels in left and right side of each anatomical area)[1]	x y z	T score	Corrected *p* value (FDR)	Cluster size (mm^3, corrected cluster level p value)[2]
Genu of corpus callosum (8)/Body of corpus callosum (102)/Anterior limb of internal capsule (R:15)/Anterior corona radiata (R:57)/Superior corona radiata (R:217)/External capsule (R:14)/Cingulum (R:10)/Superior longitudinal fasciculus (R:33)/Superior fronto-occipital fasciculus (R:2)/	16 4 38	4.77	0.006	8280, <0.001

Superior longitudinal fasciculus (R:21)/	50 −28 34	4.22	0.009	632, 0.042
Body of corpus callosum (12)/Splenium of corpus callosum (14)/Superior corona radiata (L:72)/Posterior corona radiata (L:129)/Superior longitudinal fasciculus (L:54)/	−22 −26 32	3.98	0.011	4056, <0.001
Anterior limb of internal capsule (L:18)/Anterior corona radiata (L:58)/External capsule (L:11)/	−28 20 16	3.71	0.013	888, 0.009
Posterior corona radiata (R:5)/Superior longitudinal fasciculus (R:21)/	28 −44 32	3.70	0.013	608, 0.049

[1]The anatomical labels and significant clusters of major white matter fibers were determined using the ICBM DTI-81 Atlas (http://www.loni.ucla.edu/).

[2]Only clusters that surpassed the extent threshold with voxel-level cluster-determining threshold ($P < 0.05$, corrected for the false discovery rate) were noted.

Supplemental Table S4. Brain regions exhibiting significant positive correlations between diastolic BP and MD changes in longitudinal analyses.

Included large bundles (number of significant voxels in left and right side of each anatomical area)[1]	x y z	T score	Corrected *p* value (FDR)	Cluster size (mm³, corrected cluster level p value)[2]
Positive correlation between diastolic BP and change in MD				
Genu of corpus callosum (11)/Body of corpus callosum (173)/Anterior limb of internal capsule (R:71)/Posterior limb of internal capsule (R:41)/Retrolenticular part of internal capsule (R:40)/Anterior corona radiata (R:114)/Superior corona radiata (R:329)/Posterior corona radiata (R:111)/External capsule (R:113)/Cingulum (R:43)/Superior longitudinal fasciculus (R:296)/Superior fronto-occipital fasciculus (R:10)/	10 10 30	5.25	0.001	35,408, <0.001
Genu of corpus callosum (16)/Body of corpus callosum (97)/Splenium of corpus callosum (9)/Anterior limb of	−20	4.31	0.003	37,840, <0.001

internal capsule (L:66)/Posterior limb of internal capsule (L:14)/Retrolenticular part of internal capsule (L:41)/Anterior corona radiata (L:146)/Superior corona radiata (L:244)/Posterior corona radiata (L:297)/Posterior thalamic radiation (L:34)/Sagittal stratum (L:1)/External capsule (L:61)/Cingulum (L:49)/Stria terminalis (L:4)/Superior longitudinal fasciculus (L:383)/Superior fronto-occipital fasciculus (L:8)/	−1<br>6<br>58			
Positive correlation between diastolic BP and change in RD				
Genu of corpus callosum (13)/Body of corpus callosum (182)/Splenium of corpus callosum (5)/Anterior limb of internal capsule (R:58)/Posterior limb of internal capsule (R:45)/Retrolenticular part of internal capsule (R:53)/Anterior corona radiata (R:145)/Superior corona radiata (R:448)/Posterior corona radiata (R:136)/External capsule	14<br>4<br>36	5.16	<0.001	37,424, <0.001

(R:118)/Cingulum (R:34)/Stria terminalis (R:1)/Superior				
longitudinal fasciculus (R:337)/Superior fronto-occipital				
fasciculus (R:11)/				
Genu of corpus callosum (28)/Body of corpus callosum	−2	4.49	0.001	41,288, <0.001
(113)/Splenium of corpus callosum (27)/Anterior limb of	6			
internal capsule (L:68)/Posterior limb of internal capsule	14			
(L:1)/Retrolenticular part of internal capsule (L:83)/Anterior	30			
corona radiata (L:219)/Superior corona radiata				
(L:375)/Posterior corona radiata (L:339)/Posterior thalamic				
radiation (L:35)/Sagittal stratum (L:1)/External capsule				
(L:55)/Cingulum (L:58)/Stria terminalis (L:9)/Superior				
longitudinal fasciculus (L:470)/Superior fronto-occipital				
fasciculus (L:11)/				

[1]The anatomical labels and significant clusters of major white matter fibers were determined using the ICBM DTI-81 Atlas (http://www.loni.ucla.edu/).

[2]Only the clusters that surpassed the extent threshold with the voxel level cluster determining the threshold ($P < 0.05$, corrected for the false discovery rate) were noted.

Supplemental Table S**5**. Brain regions that exhibited significant negative correlations between diastolic BP and ICVF changes in longitudinal analyses.

Included large bundles (number of significant voxels in left and right side of each anatomical area)[1]	x y z	T score	Corrected *p* value (FDR)	Cluster size (mm^3, corrected cluster level p value)[2]
Superior corona radiata (R:8)/Superior longitudinal fasciculus (R:5)/Superior fronto-occipital fasciculus (R:1)/	34 10 24	4.32	0.047	320, 0.004
None	42 −4 22	4.24	0.047	320, 0.004
None	−26 −42 42	3.96	0.047	248, 0.011

[1]The anatomical labels and significant clusters of major white matter fibers were determined using the ICBM DTI-81 Atlas (http://www.loni.ucla.edu/).

[2]Only the clusters that surpassed the extent threshold with the voxel-level cluster-determining threshold ($P < 0.05$, corrected for the false discovery rate) were noted.

Supplemental Table S6. Brain regions exhibiting significant positive correlations between diastolic BP and ISOVF changes in longitudinal analyses.

Included large bundles (number of significant voxels in left and right side of each anatomical area)[1]	x y z	T score	Corrected *p* value (FDR)	Cluster size (mm^3, corrected cluster level p value)[2]
Genu of corpus callosum (11)/Body of corpus callosum (125)/Anterior limb of internal capsule (R:27)/Posterior limb of internal capsule (R:14)/Retrolenticular part of internal capsule (R:3)/Anterior corona radiata (R:155)/Superior corona radiata (R:294)/External capsule (R:68)/Cingulum (R:26)/Superior longitudinal fasciculus (R:24)/Superior fronto-occipital fasciculus (R:4)/	12 12 30	5.75	<0.001	15,128, <0.001
Genu of corpus callosum (20)/Body of corpus	−22	5.20	<0.001	26,152, <0.001

callosum (42)/Anterior limb of internal capsule	12
(L:67)/Posterior limb of internal capsule	18
(L:2)/Retrolenticular part of internal capsule	
(L:6)/Anterior corona radiata (L:375)/Superior corona	
radiata (L:227)/Posterior corona radiata	
(L:106)/Posterior thalamic radiation (L:41)/External	
capsule (L:51)/Cingulum (L:2)/Superior longitudinal	
fasciculus (L:207)/Superior fronto-occipital fasciculus	
(L:15)/	

[1]The anatomical labels and significant clusters of major white matter fibers were determined using the ICBM DTI-81 Atlas (http://www.loni.ucla.edu/).

[2]Only the clusters that surpassed the extent threshold with the voxel level cluster determining the threshold ($P < 0.05$, corrected for the false discovery rate) were noted.

Supplemental Table S7. Comparisons of statistical results of the effects of diastolic BP with those of systolic BP.

	Effects of diastolic BP in the main analyses				Effects of systolic BP in the analyses that replaced covariates of diastolic BP with those of systolic BP			
	N	Standardized beta	T	P (uncorrected)	N	Standardized beta	T	P (uncorrected)
Fluid intelligence change	12,827	−0.002 (−0.019, 0.014)	−0.283	0.777	12,827	-0.002 (-0.019, 0.015)	-0.227	0.820
Reaction time change	37,811	−0.012 (−0.022, −0.003)	−2.478	0.013	37,811	-0.017 (-0.027, -0.007)	-3.448	0.001
Visuospatial memory (error) change	37,261	−0.004 (−0.012, 0.005)	−0.834	0.404	37,261	-0.001 (-0.009, 0.008)	-0.150	0.881
Depressive symptoms change	38,461	−0.012 (−0.021, −0.003)	−2.589	0.010	38,461	-0.009 (-0.018, 0)	-1.903	0.057
rGMV change	2274	0.045 (0.002,0.088)	2.067	0.039	2274	0.059 (0.015, 0.102)	2.644	0.008

rWMV change	2274	−0.022 (−0.065,0.021)	−0.991	0.322	2274	-0.024 (-0.068, 0.019)	-1.091	0.276
FA change	2240	−0.066 (−0.11,−0.022)	−2.922	0.004	2240	-0.029 (-0.073, 0.016)	-1.261	0.207
MD change	2240	0.055 (0.012,0.098)	2.502	0.012	2240	0.063 (0.02, 0.107)	2.857	0.004
AD change	2240	0.044 (0.002,0.087)	2.051	0.040	2240	0.065 (0.022, 0.108)	2.957	0.003
RD change	2240	0.064 (0.021,0.107)	2.933	0.003	2240	0.058 (0.014, 0.101)	2.596	0.009
ICVF change	2240	−0.05 (−0.093,−0.008)	−2.306	0.021	2240	-0.064 (-0.107, -0.021)	-2.893	0.004
ISOVF change	2240	0.043 (−0.001,0.087)	1.929	0.054	2240	0.035 (-0.01, 0.079)	1.535	0.125
OD change	2240	−0.014	−0.682	0.496	2240	-0.036	-1.699	0.089

(−0.055,0.027)	(-0.077, 0.005)

Supplemental Table S8. Comparisons of statistical results of the effects of diastolic BP with and without the inclusion of major relevant comorbidities as covariates.

	Effects of diastolic BP in the main analysis				Effects of diastolic BP in the analyses that included existence of diagnosis of 4 diseases as covariates			
	N	Standardized beta	T	P (uncorrected)	N	Standardized beta	T	P (uncorrected)
Fluid intelligence change	12,827	−0.002 (−0.019, 0.014)	−0.283	0.777	12,813	-0.002 (-0.019, 0.014)	-0.781	0.278
Reaction time change	37,811	−0.012 (−0.022, −0.003)	−2.478	0.013	37,756	-0.012 (-0.021, -0.002)	-2.357	0.018
Visuospatial memory (error) change	37,261	−0.004 (−0.012, 0.005)	−0.834	0.404	37,229	-0.003 (-0.011, 0.005)	-0.738	0.460
Depressive symptoms change	38,461	−0.012 (−0.021, −0.003)	−2.589	0.010	38,426	-0.011 (-0.020, -0.002)	-2.320	0.020
rGMV change	2274	0.045	2.067	0.039	2274	0.045	2.060	0.040

		(0.002,0.088)				(0.002, 0.088)		
rWMV change	2274	−0.022 (−0.065,0.021)	−0.991	0.322	2274	-0.023 (-0.066, 0.020)	-1.034	0.301
FA change	2240	−0.066 (−0.11,−0.022)	−2.922	0.004	2240	-0.065 (-0.109, -0.021)	-2.882	0.004
MD change	2240	0.055 (0.012,0.098)	2.502	0.012	2240	0.057 (0.014, 0.101)	2.604	0.009
AD change	2240	0.044 (0.002,0.087)	2.051	0.040	2240	0.047 (0.004, 0.090)	2.163	0.031
RD change	2240	0.064 (0.021,0.107)	2.933	0.003	2240	0.066 (0.023, 0.110)	3.016	0.003
ICVF change	2240	−0.05 (−0.093,−0.008)	−2.306	0.021	2240	-0.054 (-0.097, -0.011)	-2.457	0.014
ISOVF change	2240	0.043 (−0.001,0.087)	1.929	0.054	2240	0.043 (-0.001, 0.087)	1.938	0.053

OD change	2240	−0.014 (−0.055,0.027)	−0.682	0.496	2240	-0.017 (-0.057, 0.024)	-0.800	0.424

Reference

1. Takeuchi, H.; Kawashima, R. Diet and Dementia: A Prospective Study. *Nutrients* **2021**, *13*, Article 4500, doi:ARTN 4500 10.3390/nu13124500.

2. Townsend, P. Deprivation. *J. Soc. Policy* **1987**, *16*, 125-146.

3. Okbay, A.; Beauchamp, J.P.; Fontana, M.A.; Lee, J.J.; Pers, T.H.; Rietveld, C.A.; Turley, P.; Chen, G.B.; Emilsson, V.; Meddens, S.F., et al. Genome-wide association study identifies 74 loci associated with educational attainment. *Nature* **2016**, *533*, 539-542, doi:10.1038/nature17671.

4. Shen, X.; Cox, S.R.; Adams, M.J.; Howard, D.M.; Lawrie, S.M.; Ritchie, S.J.; Bastin, M.E.; Deary, I.J.; McIntosh, A.M.; Whalley, H.C. Resting-state connectivity and its association with cognitive performance, educational attainment, and household income in the UK Biobank. *Biological Psychiatry: Cognitive Neuroscience and Neuroimaging* **2018**, *3*, 878-886.

5. Cullen, B.; Newby, D.; Lee, D.; Lyall, D.M.; Nevado-Holgado, A.J.; Evans, J.J.; Pell, J.P.; Lovestone, S.; Cavanagh, J. Cross-sectional and longitudinal analyses of outdoor air pollution exposure and cognitive function in UK Biobank. *Scientific reports* **2018**, *8*, 1-14.

6. Sarkar, S.N.; Huang, R.-Q.; Logan, S.M.; Yi, K.D.; Dillon, G.H.; Simpkins, J.W. Estrogens directly potentiate neuronal L-type Ca2+ channels. *Proceedings of the National Academy of Sciences* **2008**, *105*, 15148-15153.

7. Howe, L.J.; Lawson, D.J.; Davies, N.M.; Pourcain, B.S.; Lewis, S.J.; Smith, G.D.; Hemani, G. Genetic evidence for assortative mating on alcohol consumption in the UK Biobank. *Nat. Commun.* **2019**, *10*, 1-10.

8. Batty, G.D.; McIntosh, A.M.; Russ, T.C.; Deary, I.J.; Gale, C.R. Psychological distress, neuroticism, and cause-specific mortality: early prospective evidence from UK Biobank. *J. Epidemiol. Community Health* **2016**, *70*, 1136-1139.

9. Khubchandani, J.; Brey, R.; Kotecki, J.; Kleinfelder, J.; Anderson, J. The psychometric properties of PHQ-4 depression and anxiety screening scale among college students. *Arch. Psychiatr. Nurs.* **2016**, *30*, 457-462.

10. Miller, K.L.; Alfaro-Almagro, F.; Bangerter, N.K.; Thomas, D.L.; Yacoub, E.; Xu, J.; Bartsch, A.J.; Jbabdi, S.; Sotiropoulos, S.N.; Andersson, J.L. Multimodal population brain imaging in the UK Biobank prospective epidemiological study. *Nat. Neurosci.* **2016**, *19*, 1523-1536.

11. Alfaro-Almagro, F.; Jenkinson, M.; Bangerter, N.K.; Andersson, J.L.; Griffanti, L.; Douaud, G.; Sotiropoulos, S.N.; Jbabdi, S.; Hernandez-Fernandez, M.; Vallee, E. Image processing and Quality Control for the first 10,000 brain imaging datasets from UK Biobank. *Neuroimage* **2018**, *166*, 400-424.

12. Ashburner, J.; Friston, K.J. Voxel-based morphometry-the methods. *Neuroimage* **2000**, *11*, 805-821.

13. Daducci, A.; Canales-Rodríguez, E.J.; Zhang, H.; Dyrby, T.B.; Alexander, D.C.; Thiran, J.-P. Accelerated microstructure imaging via convex optimization (AMICO) from diffusion MRI data. *Neuroimage* **2015**, *105*, 32-44.

14. Takeuchi, H.; Taki, Y.; Hashizume, H.; Asano, K.; Asano, M.; Sassa, Y.; Yokota, S.; Kotozaki, Y.; Nouchi, R.; Kawashima, R. Impact of videogame play on the brain's microstructural properties: Cross-sectional and longitudinal analyses. *Mol. Psychiatry* **2016**, *21*, 1781-1789.

15. Takeuchi, H.; Taki, Y.; Thyreau, B.; Sassa, Y.; Hashizume, H.; Sekiguchi, A.; Nagase, T.; Nouchi, R.; Fukushima, A.; Kawashima, R. White matter structures associated with empathizing and systemizing in young adults. *Neuroimage* **2013**, *77*, 222-236.

16. Inouye, M.; Abraham, G.; Nelson, C.P.; Wood, A.M.; Sweeting, M.J.; Dudbridge, F.; Lai, F.Y.; Kaptoge, S.; Brozynska, M.; Wang, T. Genomic risk prediction of coronary artery disease in 480,000 adults: implications for primary prevention. *J. Am. Coll. Cardiol.* **2018**, *72*, 1883-1893.

17. Dufouil, C.; de Kersaint–Gilly, A.; Besancon, V.; Levy, C.; Auffray, E.; Brunnereau, L.; Alperovitch, A.; Tzourio, C. Longitudinal study of blood pressure and white matter hyperintensities: the EVA MRI Cohort. *Neurology* **2001**, *56*, 921-926.

18. Imaizumi, T. The origin and clinical importance of white matter lesion. *Japanese Journal of Stroke* **2016**, *38*, 69-76.

19. Warnert, E.A.; Rodrigues, J.C.; Burchell, A.E.; Neumann, S.; Ratcliffe, L.E.; Manghat, N.E.; Harris, A.D.; Adams, Z.; Nightingale, A.K.; Wise, R.G. Is high blood pressure self-protection for the brain? *Circ. Res.* **2016**, *119*, e140-e151.

20. Semplicini, A.; Maresca, A.; Boscolo, G.; Sartori, M.; Rocchi, R.; Giantin, V.; Forte, P.L.; Pessina, A.C. Hypertension in acute ischemic stroke: a compensatory mechanism or an additional damaging factor? *Arch. Intern. Med.* **2003**, *163*, 211-216.
